# Hospital and Institutional News

**Published:** 1918-02-09

**Authors:** 


### HOSPITAL AND INSTITUTIONAL NEWS.

### A CHAIR OF PREVENTIVE MEDICINE.

The Welsh National Medical College at Cardiff has been offered a sum of £30,000 by Miss Talbot to endow a chair of preventive medicine. The income thus provided should be sufficient to attract a man with the highest qualifications, and the School of Preventive Medicine, as perhaps it will be called, has been offered the old offices of the Glamorgan County Council, in Westgate Street. To provide a subject with a chair is less objectionable than to provide it with a Cabinet Minister, and we hope that the future professor will be able to co-ordinate the diverse aspects of his subject, and will not forget to teach that sanitation and public hygiene have probably done more, and are certainly freer from objectionable features, than whatever the latest fashion of immunisation by injections happens to be. The municipal dustbin does not sound so pretentious as the research laboratory, but it is an open question whether it and the sanitation which it represents have not been equally valuable in the maintenance of public health.

### THE MEMORIAL BUILDINGS AT LEEDS.

The King Edward Memorial Extension to Leeds General Infirmary, for which £120,000, or four-fifths of the total, has been raised, is being put to such gradual use that it is well from time to time to note the occupation of the new buildings. Some two-thirds have been finished, the remainder awaits the end of the war. Of the four new blocks, that devoted to the nurses, who now have separate bedrooms, has been occupied for a year or so. Of the three new ward blocks, the central one with its three storeys alone has been built, and at present some 100 soldiers are under treatment there. The theatre block, containing five theatres on the top storey, and consulting-rooms for out-patients upon the ground floor, is ready except for the installation of electric light. The ophthalmic theatre has been endowed by Ranjitsinhji with 100 guineas a year "in thankful remembrance of his recovery from a shooting accident, in August 1915." If the Maharaja's mishap had occurred on the cricket field there would have been an even closer association in English memory between the patients who will see the tablet recording this gift and the once well-known and popular cricketer. By means of the new consulting-rooms for out-patients, all the General Infirmary's out-patients can now be treated in its own walls, and the hospitality of the Women and Children's Hospital is no longer necessary.

### THE NEW SUPERVISION OF ORTHOPÆDIC CASES.

No more pressing question has arisen than the skilled treatment and supervision of military orthopædic cases. An Army Council Instruction, aimed apparently at the better control of such patients, is therefore worth attention. It lays down that in future all the military orthopædic cases which are transferred to this country will be sent direct to a military orthopædic centre, or at least to a hospital which has been approved for dealing with such men. In future, the instruction proceeds, these patients will not be distributed from railway stations to auxiliary hospitals. Centres for the treatment of orthopædic cases have been now established at a number of hospitals in the various commands throughout the kingdom, where special wards for officers are provided. Whether this number is, in fact, sufficient is perhaps not so certain. But at all events the organisation is to be improved, and an orthopædic case is not in future to be sent to any place where a bed can be found for him. Australian, Canadian, and New Zealand patients of this type are to be dealt with as before.

### A BIG PROPOSAL AT BRADFORD.

Speaking at the recent meeting of the Bradford Hospital and Convalescent Fund, Mr. Herbert Gill raised the question what memorial Bradford would raise at the end of the war. He added that he hoped that the city would not let the chance slip by, and expressed the opinion that no more suitable general memorial could be found than a proper general infirmary. The condition of the present hospital has given rise to heart-searchings before now among those who know its limitations, and realise the place which it ought to fill in the life of the city. Therefore, Mr. Gill is right to raise the question again, and to use the meeting of the Hospital Fund as a platform from which to put forward a scheme much more important than the immediate matter to be considered.

### "P STANDS FOR ——"

If we continually remark on the appeals employed to raise money on behalf of King Edward VII.'s Hospital, Cardiff, it is not so much because they are new, as that they are applied amusingly, persistently, and methodically. All hospital secretaries write letters to the local newspapers, but Mr. Rea writes almost every day. All hospitals send sometimes a grateful letter for publication: Mr. Rea, collecting a few specimens, writes what is virtually an essay on "grateful letters," and is overwhelmed with a new list of subscriptions, and a new supply of letters the next morning. Instead of "venturing to appeal to the hospitality of your columns," he comes out boldly as one of the most varied and original writers on the Cardiff press. Unfailing in resource he is always interesting, and we expect to see him in charge of a regular "Hospital column" by and by. The latest example of his method is to acknowledge the receipt of £205 from a committee of residents at Pontyclun by publishing beneath it the number of patients from this district treated at the hospital, and the amount of subscriptions received from it. But, unlike the average secretary, he does not stop there. For, after pointing out that the new donation is redressing an adverse balance in the past against the district, Mr. Rea merrily goes on: "Let us try a few more P's." He gives a list of eleven more districts with this initial letter, and sets in double column the subscriptions received, and the cost of patients treated, from each. When all the P's know the state of their account with the hospital, Mr. Rea is confident that they will keep their accounts square, and then he inimitably adds, "perhaps later we may try other letters of the alphabet." Could anything be better or more neatly done? Method is not the dull dog which it is supposed to be by those who take it for routine. Method is the tortoise which wins the race, and though the battle may not be always to the strong, the race is Mr. Rea's in Cardiff.

### THE PORTHKERRY BED.

A fine example has been set by the seaside hamlet of Porthkerry, near Barry, the residents of which—a mere handful when all told—have contributed 1,000 guineas for the endowment of a "Porthkerry Bed" at King Edward VII.'s Hospital, Cardiff. The idea was taken up and carried through to success within a space of less than seven weeks by an enthusiastic young lady, who was seconded by the warm interest of her parents. The subscriptions were limited to persons resident in the hamlet, and the result is, as Mr. Leonard Rea, the hospital secretary, puts it, that "Porthkerry will now provide accommodation for some seventeen sick persons each and every year henceforth and for ever." This is a strong challenge to a thousand-and-one small places to show in a similar way their appreciation of the hospital which serves their neighbourhood.

### AN OFFICER'S MUNIFICENCE.

Colonel C. H. Innes Hopkins, of Newcastle, has shown a twofold generosity in his gift to the nation of "The Towers," Ryton. While he has given it to the Ministry of Pensions, for use as an orthopædic hospital for soldiers disabled in the present war, he has provided that, when the premises cease to be required for this purpose, they are to revert to the Newcastle Royal Victoria Infirmary. The gift is a memorial to his two sons, Charles and Gastell, both of whom have been killed in the war. "The Towers," originally the home of the McIntyres, was bought by Colonel Innes Hopkins in 1901, and is a roomy house with pleasant grounds, which the donor hopes will be preserved in their present condition. When adapted it will accommodate a considerable number of patients.

### AN ARGUMENT TO ANNUAL SUBSCRIBERS.

The replacement of a casual cheque by a subscription at so much per head of the number of a subscriber's employees has produced such satisfactory results in the Midlands that an application of a similar argument to annual subscribers is worth noting. A provincial hospital chairman lately pointed out that annual subscriptions are based more on custom than on principle. The typical subscription was two guineas. This sum, he remarked, provided for a stay of eight days. But the average stay of a patient was thirty days, and, therefore, if these subscriptions were to express a general principle, subscribers might be induced to raise them to six guineas. It may be hard to induce many people so to raise them, and some cannot afford to do so. But we are sure that an attempt is more likely to be successful which adopts an argument that appeals to the reason of the donor, and allows him a comprehension of the financial position, in which he can shoulder a responsible part, and feel that he is doing a perfectly definite service with his money.

### HOSPITAL SATURDAY FUND GRANTS.

Grants amounting to £45,443 were made to the medical charities at a recent special meeting of the board of delegates of the Hospital Saturday Fund (London). Two hundred and thirty-one institutions—five more than the previous year—participated. The awards exceeded those for 1916 by £2,177. Sir Vezey Strong (the Chairman of the Fund), who presided, commented upon the highly satisfactory character of the collection, and trusted that the result of last year's effort would be maintained when peace was declared.

### CARD GAMES IN CONVALESCENCE.

The Headquarters Organising Clothing Committee of the Scotch Branch of the British Red Cross Society, in issuing an appeal for special gifts, has asked for a supply of card-tables. These card-tables are destined for a hospital for mental cases. Whether they are asked for as part of the general equipment of this hospital we do not know, but the request suggests that card games are found to be peculiarly grateful to mental cases. It is perhaps easier to maintain the interest of an overtaxed mind upon a game of cards than upon any other occupation. The hands are employed, which is always desirable, and in this way the brain is less taxed than in the seemingly less exacting task of reading. Most men who are not cripples do not take kindly to knitting, which is known to benefit women whose nerves have been overtaxed by illness or after an operation.

### THREE AUTHORITIES POWERLESS BEFORE AN EPIDEMIC.

The epidemic of measles which has occurred in Leicester has led to some criticism by the medical officer of health and by the school medical officer on the ground that their present powers discourage them from tackling the problem successfully. Since the epidemic has prevailed for three months the matter is serious. It is remarked that the compulsory notification of measles in itself accomplishes nothing but a statistical record, and that the Sanitary Committee are under the control of the education authorities. Even if the schools were closed with the latter's consent, such preventive measures as isolation are not effectively taken, and the cry for more compulsion is raised. We do not believe in compulsion as a panacea for this or other evils. It has surely been found out in the past three years. A simpler method would be co-ordination between the three authorities concerned. If that were willingly given we think that notification, by making public the extent of the danger, might enable the authorities to take those preventive methods which notification was intended to precede.

### WORKING UNDER DIFFICULTIES.

Some idea of the difficulties at present experienced in infirmaries is shown in a report presented to the Lambeth Board of Guardians by Dr. Lionel Baly, the medical superintendent at their infirmary. During the week after Christmas the number of patients increased rapidly, rising to over a thousand. At the same time an outbreak of measles of a severe type occurred. Dr. Baly appealed to the Metropolitan Asylums Board, but, owing to their lack of staff, no assistance could be given. At the date of his report forty-three cases had developed, of whom ten had died. There were 299 children in the infirmary, and as most of the female wards had been infected the children could not be admitted there. Two fair-sized wards had to be reserved for measles and whooping-cough. Some slight relief had been obtained by the Southwark Board of Guardians, whose patients were under treatment at Lambeth, receiving sixty patients and transferring them to their Newington institution. The Guardians have requested their clerk to write a strong letter of protest to the Local Government Board with regard to the failure of the Metropolitan Asylums Board to deal with the cases for which they are responsible.

### THE BURIED SIDE OF HOSPITAL LIFE.

The letter from the Church Missionary Society which we publish in another column is welcome, because it shows that the Church Missionary Society appreciates the advantage of keeping the world informed of the doings of its missionary hospitals. We were grateful for the information lately supplied by the society in connection with Gaza Hospital. The point, however, is that the doings of these mission hospitals are virtually buried, and we have always been of opinion that if their work were made better known by well-informed and illustrated articles the public would take a less languid interest in mission work than is at present the case. When some stray hint reaches us, as it does from time to time (witness a recent article in The Hospital on the mission hospital in Korea), we gladly publish it, because this side of hospital work abroad is as little known as it is full of interest. Such information, however, is never volunteered spontaneously by the missionary societies, nor do they seem to realise that to assist and foster publicity in this regard is an important part of their duty. On behalf of the public we should welcome indeed regular information "of important developments in medical missionary work." In fact, we should prefer to be kept in constant personal touch with the lives and doings of these, perhaps the most unexceptionable workers in the whole field of missions. It is for the Church Missionary Society to give us the opportunity for which we have long waited.

### AFTER-CARE OF CONSUMPTIVES.

West Ham Corporation has prepared a scheme for the care and after-care of persons suffering from tuberculosis. The objects are to support and assist measures for the prevention and cure of consumption, and for this purpose use will be made of existing agencies which have for their object the restoration of individual consumptives to health and independence. The committee to be formed will comprise the Mayor, four Town Councillors, four members of the Insurance Committee, and two members of the Panel and local Medical Committee. The medical officer of health, the medical superintendent of the West Ham Sanatoria, the medical superintendent of the infirmary, and the local tuberculosis officer will be *ex-officio* members. The committee will be elected annually, and be empowered to co-opt others who are willing to be active members. The committee will, where possible, arrange for the investigation of the home and social conditions of the patients when required, with a view to ascertaining their bearing on the question of the most effective assistance which can be given to the patients by the committee. A panel of voluntary workers may be formed, and a system of visitation by the persons on such list worked in conjunction with the committee. During the period tuberculous persons are undergoing home or dispensary treatment the committee will endeavour to make suitable arrangements for the proper home conditions and for the provision, if necessary, of all subsidiary aids to the treatment of such persons, as nourishment, clothing, special appliances, conveyance to hospital or surgical-instrument makers, shelter accommodation, suitable employment, and advice generally.

### HOSPITAL EMPLOYEE CHARGED.

In connection with certain charges of theft made against an electrician named Austin, William Robert Cooper, 53, surgery assistant at St. Thomas's Hospital, was charged at Westminster Police Court a fortnight ago with stealing certain stores and instruments from the hospital. On finding at Austin's house, among stolen property, certain instruments and goods which included some identified as the property of St. Thomas's Hospital, Cooper was arrested. Evidence was given by Mr. G. Q. Roberts, secretary of St. Thomas's Hospital, who declared that Cooper had been a trusted servant at the hospital where he had been employed for thirty-three years; he was a man of considerable skill and technical knowledge. Austin, added Mr. Roberts, had long been a friend of Cooper, and was the cause of his present misfortune. After sentencing Austin to twelve months' imprisonment with hard labour, the magistrate, Mr. Francis, remanded Cooper for a week, having remarked that he had been misled by the evil influence of his friend and has since accepted Cooper's own recognizances to come up for judgment if called on.

### HOSPITAL FOR TUBERCULOUS CHILDREN.

In the West Riding the medical inspection of school children reveals on an average every year 150 cases of pulmonary and 210 cases of other forms of tuberculosis. The County Council has accordingly been discussing the matter with the Education Committee, and now the Health Committee of the County Council reports that to the passers-by in the streets of the West Riding there is abundant evidence that a large number of cases of tuberculous disease of bones and joints have not received appropriate treatment, and the need for a special hospital is very urgent. Many persons who go through life crippled and deformed might have been useful workers if suitable treatment had been available at the proper time. Hence it is proposed to prepare plans for such a sanatorium to be erected at the end of the war. The example of Alton shows how much good can be done in a special tuberculosis hospital.

### THE PROBLEM OF DEPLETED STAFFS.

REPORTS of local authorities throughout the country indicate that attention is being paid to problems associated with the depletion of staffs. In Leeds there is a proposal to organise lectures for the training of visitors to mentally defectives, and the Halifax Corporation has arranged to send its inspectors to the lectures, if they are arranged. The president of the Leeds Maternity Hospital has suggested a conference of voluntary and official workers in connection with maternity and infant welfare, and the Leeds Corporation has agreed to support the conference. The Association of Municipal Corporations is circularising authorities with regard to the care of mental defectives in their own homes in view of the shortage of institution accommodation, and asking how far it has been possible for statutory authorities to employ Public Health visitors to assist in the supervision of defectives.

### THE RETENTION OF A SECRETARY.

SOUTHWARK TRIBUNAL has been called upon to decide whether the committee of the Hospital for Skin Diseases, Blackfriars Road, should be allowed to retain the services of its secretary, Mr. F. Hayter, who is thirty-two years of age, and placed in Grade 3. Captain Owen Davies, R.A.M.C., and Dr. Abercrombie both bore testimony to the work done by Mr. Hayter, who, in addition to his secretarial duties, is operating the *x*-rays and radium treatment, as well as undertaking pathological and bacteriological labours in the laboratory. Over 350 out-patients attend at the hospital every week, and six beds are set aside for the special treatment of venereal disease. Captain Davies, who is under orders to proceed to Palestine, urged that, in his absence, Mr. Hayter is the only person capable of doing the clerical work. In the event Mr. Hayter's previous conditional exemption has been withdrawn, and six months' exemption has been granted to him.

### MEN FOR THE V.T.C.

NEW instructions have just been issued to local and appeal Tribunals regarding the conditions under which they should impose an obligation to join the Volunteer Force upon men exempted for business and other reasons. In cases where the work the man is performing is exhausting physically, the ordinary drills may be excused, and if the state of the man's health does not permit him to carry out the routine of training, other forms of National Service may be imposed, such as special constable duty, Red Cross work, or service with the Fire Brigade, even, indeed, some form of land cultivation. The vexed question of dentists and their exemption is solved in an eminently practical manner by decreeing that where an exemption has been granted, the Tribunal shall have the power to impose such professional service as may be recommended by the Dental Service Committee. It is interesting in this connection to note that medical students, hitherto totally exempt after the intermediate examination had been passed, are now liable to be called up for six months' service with the Navy as surgeon probationers, after which they will be released to complete their course of study.

### THE LATE PROFESSOR REMINGTON.

BY the death of Professor Remington, of the Philadelphia College of Pharmacy, news of which has just reached us, America has lost its most distinguished authority on drugs. Among medical practitioners and pharmacists Professor Remington had a world-wide reputation, his book on the "Practice of Pharmacy," which has run through many editions, being generally regarded as the textbook on pharmacy in the English language. He was best known, however, as Chairman of the Committee of Revision of the United States Pharmacopœia. In America the method of producing and revising the Pharmacopœia differs entirely from the English system, as witnessed by the fact that the Chairman of the Committee of Revision was a pharmacist and not a medical man. Professor Remington was not an infrequent visitor to Europe; on the occasion of his last visit to London the British Pharmacopœia was nearing the end of the process of revision, and he took the opportunity of exchanging notes with its editors.

### THIS WEEK'S DRUG MARKET.

PRICES are firmly maintained for practically all drugs, and failing the institution of any system of fixed maximum prices it seems more than probable that values will continue to move upwards. America, on which we depend for such a large proportion of our drug supply, is sending us less and less, and consequently the shortage is becoming much more pronounced. There is no relief in the shortage of potassium and ammonium bromides, but the supply of sodium bromide appears to be adequate. A new outlet has been found for our limited supplies of saccharin; this substance is now being served instead of sugar at many of the popular tea-shops, and if this practice continues prices are likely to be maintained at a high level. Acetyl salicylic acid is offered at what might be considered rather low rates. Acetanilide and hexamine continue scarce, and the prices are well maintained. The shortage of the supply of camphor is pronounced, and a further advance in price is not improbable.

